# Cobalt-Containing Nanoporous Nitrogen-Doped Carbon Nanocuboids from Zeolite Imidazole Frameworks for Supercapacitors

**DOI:** 10.3390/nano9081110

**Published:** 2019-08-02

**Authors:** Yu Song, Mingyue Zhang, Tianyu Liu, Tianjiao Li, Di Guo, Xiao-Xia Liu

**Affiliations:** 1 Department of Chemistry, Northeastern University, Shenyang 110819, China; 2 Department of Chemistry, Virginia Tech, Blacksburg, VA 24060, USA

**Keywords:** metal–organic frameworks, zeolite–imidazole frameworks, cobalt, carbon, nanoporous, supercapacitors

## Abstract

Pyrolyzing metal–organic frameworks (MOFs) typically yield composites consisting of metal/metal oxide nanoparticles finely dispersed on carbon matrices. The blend of pseudocapacitive metal oxides and conductive metals, as well as highly porous carbon networks, offer unique opportunities to obtain supercapacitor electrodes with mutually high capacitances and excellent rate capabilities. Herein, we demonstrate nitrogen-doped carbon nanocuboid arrays grown on carbon fibers and incorporating cobalt metal and cobalt metal oxides. This composite was synthesized via pyrolysis of a chemical bath deposited MOF, cobalt-containing zeolite imidazole framework (Co–ZIF). The active materials for charge storage are the cobalt oxide and nitrogen-doped carbon. Additionally, the Co metal and the nanoporous carbon network facilitated electron transport and the rich nanopores in each nanocuboid shortened ion diffusion distance. Benefited from these merits, our Co–ZIF-derived electrode delivered an areal capacitance of 1177 mF cm^−2^ and excellent cycling stability of ~94% capacitance retained after 20,000 continuous charge–discharge cycles. An asymmetric supercapacitor prototype having the Co–ZIF-derived hybrid material (positive electrode) and activated carbon (negative electrode) achieved a maximal volumetric energy density of 1.32 mWh cm^−3^ and the highest volumetric power density of 376 mW cm^−3^. This work highlights the promise of metal–metal oxide–carbon nanostructured composites as electrodes in electrochemical energy storage devices.

## 1. Introduction

Developing nanomaterials for electrochemical energy storage is among the forefront of materials research [1,2,3,4,5]. A plethora of nanostructured transition metal oxides, for example, manganese dioxide (MnO_2_) [6,7,8], iron oxide (Fe_2_O_3_) [9,10,11], molybdenum trioxide (MoO_3_) [12,13], tungsten oxide (WO_3_) [14], and cobalt oxides (Co_3_O_4_ and CoO) [15,16], are potent electrode materials for secondary batteries and supercapacitors, thanks to their characteristics of high theoretical capacities or capacitances, earth abundance, low toxicity, and cost-effectiveness. One major challenge of these nanomaterials for supercapacitors is that their experimental capacities or capacitances are far below the theoretical values because the electrically insulating or semiconducting natures dramatically impede fast electron transport and ion diffusion, which are the two prerequisites for excellent rate capabilities and power densities [17,18,19].

Strategies to mitigate the intrinsic drawbacks of metal oxide electrodes involve two means. The first method is to incorporate metal oxides into highly conductive scaffolds, such as carbon and metals, to form composite electrodes. For example, Li et al. sandwiched a thin Mn metal layer between two MnO_2_ layers for capacitive charge storage [20]. The metal Mn central layer served as an electrically conductive substrate to remedy the negative impact of the poor electrical conductivity of MnO_2_. The second strategy is to construct hierarchically porous architectures with pore sizes spanning from sub-nanometers to hundreds of micrometers [21]. These unique architectures are beneficial to speed up ion diffusion, facilitate electrolyte ion infiltration as well as enhance active surface areas [22,23]. Song and Liu et al. devised a glucose-assisted hydrothermal method to fabricate mesoporous iron(III) oxide films with tunable pore sizes [9]. With 1 µm in thickness and 5 mg cm^−2^ in mass loading, the mesoporous hematite electrode reached 1502 mF cm^−2^ at 1 mA cm^−2^, approximately two to three times higher than other hematite materials with similar mass loadings.

We turned our attention to metal–organic frameworks (MOFs), a family of crystalline coordination compounds consisting of organic ligands and metal nodes [24,25], to address the bottlenecks above for metal-oxide-based supercapacitor electrodes. These nanomaterials, upon thermal annealing, produced metal or metal oxide nanoparticles (from metal nodes) uniformly embedded in carbon matrices (from organic ligands). This strategy to fabricate metal–carbon composites has advantages of dispersion uniformity compared to evaporation/sputtering [26] or electrodeposition [27]. This configuration is beneficial for high-performance charge storage: The electrically conductive carbon networks, as well as metal nanoparticles, alleviate the limited electrical conductivity of metal oxides. The high porosities inherited from MOFs allow for rapid electrolyte infiltration and ion diffusion to ensure excellent power densities. The uniformly distributed metal oxide nanoparticles sustain large contact areas with electrolyte ions and maintain high capacitances and energy densities.

In this work, we synthesized a cobalt-containing MOF, termed zinc-imidazole framework-67 (Co–ZIF), as a starting material through chemical bath deposition on carbon cloth fibers. Utilizing facile thermal annealing under N_2_ atmosphere, we converted the Co–ZIF nanocuboids into nanoporous, N-doped carbon nanocuboids supporting finely dispersed cobalt metal and cobalt oxide nanoparticles. The metal Co and N-doped carbon provide electron transport expressways, and the nanopores in each nanocuboid ensure low-impedance ion diffusion. As a result, the hybrid material achieved an areal capacitance of 1200 mF cm^−2^ at 1 mA cm^−2^, and highly stable performance with more than 90% capacitance maintained after 20,000 charge–discharge cycles. Coupled with activated carbon (a negative electrode), our composite material is also a potent positive electrode of an asymmetric supercapacitor prototype.

## 2. Materials and Methods

### 2.1. Materials

Carbon cloth was obtained from USA Fuel Cell Earth Company (Woburn, MA, USA). Concentrated nitric acid (HNO_3_), cobalt nitrate hexahydrate [Co(NO_3_)_2_·6H_2_O], 2-methylimidazole, *N*-methyl-2-pyrrolidone (NMP), polyvinylidene fluoride (PVDF), activated carbon (AC), and potassium hydroxide (KOH) were all provided by Sinopharm Chemical Reagent Co., Ltd. (Shenyang, China), and used as received.

### 2.2. Fabrication of Hybrid Electrode Materials

First, a piece of carbon cloth (1 × 2 cm^2^) was hydrothermally heated at 120 °C for 2 h in a Teflon-lined autoclave containing a 30 wt.% HNO_3_ aqueous solution to increase the surface hydrophilicity. Second, 1.3 g 2-methylimidazole and 0.582 g Co(NO_3_)_2_·6H_2_O were dissolved in 50 mL deionized water stirred at room temperature for 30 min to form a clear blue solution. Third, the treated carbon cloth was soaked in the solution at 35 °C for 1 h. Afterward, the carbon cloth was washed with alcohol and deionized water to remove solution residues, and dried in a vacuum oven at 50 °C for 10 h. Last, the carbon cloth was annealed in a tube furnace at 350 °C, 450 °C, or 550 °C for 1 h under N_2_ with a ramp rate of 5 °C min^−1^, leading to electrodes denoted as Co–ZIF-X, where X = 350, 450, and 550 representing the thermal annealing temperature in °C.

### 2.3. Assembly of Asymmetric Supercapacitors

The practical electrochemical performances of the Co–ZIF-450 electrodes were evaluated in asymmetric supercapacitor prototypes possessing the Co–ZIF-450 positive electrode and an AC negative electrode. The AC negative electrode was prepared by uniformly mixing 2 g AC, 0.025 g acetylene black, and 0.8 g NMP in an agate mortar for 2 h. The resultant black slurry was spread onto a piece of carbon cloth current collector. After soaking the positive and negative electrodes in 2 M KOH aqueous solution for 10 min, we sandwiched a piece of filter paper absorbed with the KOH solution between the electrodes. Afterward, the supercapacitor was wrapped and sealed by parafilm to avoid electrolyte leakage. The volume of the asymmetric supercapacitor, including the parafilm packing, was ~0.9 mm^3^ (10 mm × 10 mm × 0.09 mm).

### 2.4. Characterizations

Carl Zeiss Ultra Plus scanning electron microscopy (SEM, Carl Zeiss, Germany) imaged the morphologies. Fourier transform infrared spectroscopy (FTIR) was conducted by an infrared spectrometer (VERTEX70, Bruker Optics, Billerica, MA, USA). X-ray diffraction (XRD) was performed by a (PANalytical X’Pert Pro, PANalytical Inc., Netherlands) X-ray powder diffractometer. X-ray photoelectron spectroscopy (XPS) were collected by an XPS spectrometer (ESCALAB 250Xi, Thermo Scientific Inc., Waltham, MA, USA) with Al *K*_α_ radiation as the source of excitation. The binding energies of the XPS data were calibrated using C 1*s* peak at 284.6 eV as the reference. Electrochemical performances of the electrodes were investigated by a VMP3 multichannel electrochemical workstation with a three-electrode testing configuration using the material of interest as the working electrode, a Hg/HgO electrode as the reference electrode, and a piece of carbon cloth as the counter electrode. The details of the methylene blue (MB) adsorption experiments for surface area determination were included in the discussion associated with Figure S3 in the Supplementary Materials.

## 3. Results and Discussion

**Figure 1:**
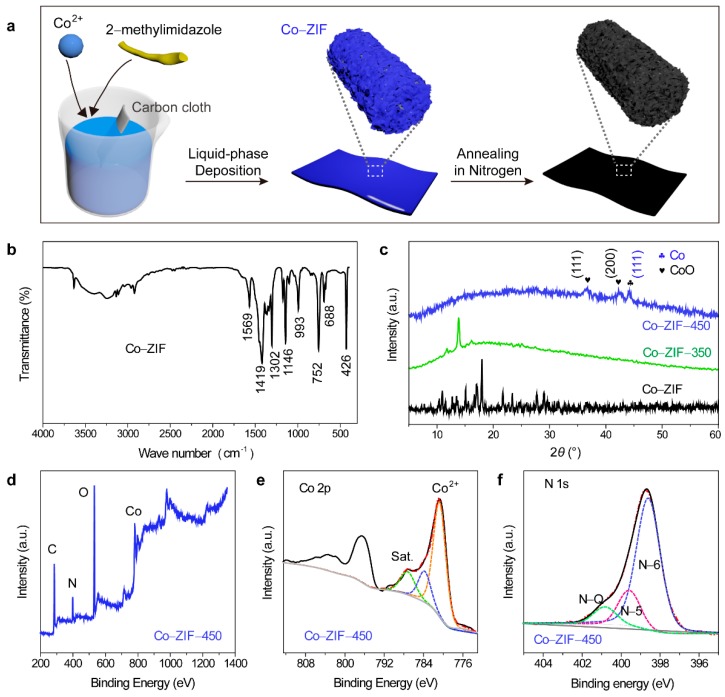
(**a**) Schematic illustrating the synthesis steps of cobalt-containing zeolite imidazole framework (Co–ZIF-X; X=thermal annealing temperature) on carbon cloth substrate. (**b**) FTIR spectrum of Co–ZIF. (**c**) XRD patterns of Co–ZIF, Co–ZIF-350, and Co–ZIF-450. (**d**) XPS survey spectrum of Co–ZIF-450. (**e**) Co 2*p* XPS spectrum of Co–ZIF-450. (**f**) N 1*s* spectrum of Co–ZIF-450. For the XPS spectra, the solid black lines are experimental data, the solid gray lines are baselines, and the dashed lines are deconvoluted peaks.

We utilized a chemical bath deposition technique to grow cobalt-containing ZIF-67 (Co–ZIF) sheets onto commercial carbon cloth, and annealed the coated carbon cloth under N_2_ to convert Co–ZIF to cobalt- and cobalt oxide-incorporated porous carbon electrodes (Figure 1a). In the FI-IR spectrum of Co–ZIF (Figure 1b), the peaks between 400 and 1700 cm^−1^ are the fingerprints of Co–ZIF [28]: The main vibration modes at 426, 1146 and 1302, and 1569 cm^−1^ correspond to the Co–N stretching, C–H vibration, and C=N vibration, respectively [29]. Additionally, the XRD pattern of Co–ZIF (Figure 1c) matched with the standard one [28], confirming the successful growth of Co–ZIF. Annealing Co–ZIF at 350 °C for 1 h partially decomposed its ligands to amorphous carbonaceous compounds, as proven by the broad diffraction peak spanning from 10° to 30°. Further elevating the annealing temperature completely converted Co–ZIF to cobalt-based compounds and amorphous carbon. While the broad peak of amorphous carbon was still present, there were three new peaks at ~36.5°, 42.4°, and 44.4°. The first two peaks were from the (111) and (200) planes of CoO (JCPDS #09-0402), and the last one was the diffraction peak of the Co (111) plane (JCPDS #01-1259). Furthermore, we used the Scherrer equation to evaluate the crystal sizes of CoO and Co:

(1) $$D=\frac{K\lambda }{\beta \left(\theta \right)\cos(\theta )}$$

where *D* is the crystal size; *K* is the Scherrer’s constant (0.89); *λ* is the wavelength of the X-ray (0.15405 nm); *θ* is the diffraction angle, and *β*(*θ*) is full width at half maximum (FWHM) of the X-ray diffraction peak (*θ*). The crystal size of CoO in Co–ZIF-450 calculated based on the (111) diffraction peak of CoO (JCPDS #09-0402) is ~10.7 nm, while that of Co nanoparticles based on the (111) diffraction peak of Co (JCPDS #01-1259) is ~17.8 nm.

XPS probed the elemental composition of the product (Co–ZIF-450) after the 450 °C annealing. The survey spectrum disclosed that Co–ZIF-450 contained elements of C, O, N, and Co (Figure 1d). We believed that the nitrogen element must come from the nitrogen-doped carbon matrix because XRD showed no cobalt nitride. The main peak at ~780.1 eV in the Co 2*p* XPS peak was from CoO [28], while the peaks at 776.1 eV and 783.9 eV presented the existence of Co metal and Co_3_O_4_, respectively (Figure 1e) [30]. The strong peak of CoO marked that the Co^2+^ ions in Co–ZIF were mostly oxidized to low-valence-state compound, CoO in the air, a phenomenon also observed in other works [30,31,32]. In addition, three synthetic peaks, pyrrolic N (N-5), pyridinic N (N-6), and quaternary N (N-Q) constituted the N 1*s* peak of Co–ZIF-450, again proving the N doping nature of the carbonaceous matrix since no peaks of Co-N bonds appeared. These N-moieties are known to offer pseudocapacitance and facilitate electron transfer at electrode/electrolyte interfaces [33].

**Figure 2:**
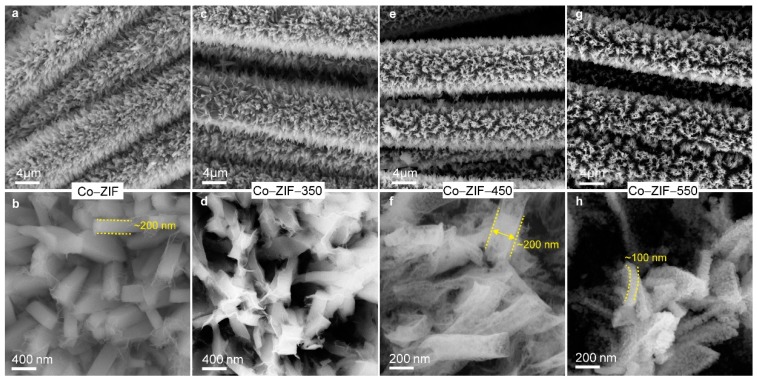
SEM images of (**a**,**b**) Co–ZIF; (**c**,**d**) Co–ZIF-350; (**e**,**f**) Co–ZIF-450; (**g**,**h**) Co–ZIF–-550. The dashed yellow lines highlight the thicknesses of corresponding nanocuboids.

We utilized SEM to investigate how the morphology evolved before and after the thermal annealing. Co–ZIF was composed of nanocuboids uniformly grown on carbon cloth fibers (Figure 2a). Each nanocuboid was ~200 nm thick (Figure 2b) and ~2 µm long (Figure S1, Supplementary Materials). Annealing Co–ZIF at 350 °C showed little deviation in morphology from its original state. The decomposition of the organic ligands of Co–ZIF perforated each nanocuboid when the annealing temperature was elevated to 450 °C and 550 °C. Additionally, the neighboring nanocuboids of Co–ZIF-550 aggregated into bundles, which could have resulted from structural collapse during the annealing step. The aggregation is unfavorable for rapid charge storage since it elongates the distances of electron transport and ion diffusion [34]. For all cases, the SEM images showed no large particles and proved that cobalt and cobalt oxide were finely dispersed without severe agglomerating into SEM-detectable particles. The fine dispersion was also evidenced by the uniform element distributions unveiled by the elemental mappings of SEM (Figure S2, Supplementary Materials).

Methylene blue adsorption experiment gauged the surface areas of Co–ZIF-350, Co–ZIF-450, and Co–ZIF-550 at room temperature. Using the absorption at 650 nm of the dye-desorbed samples (Figure S3, Supplementary Materials), we evaluated that the surface area of Co–ZIF-450 reached 61.6 m^2^ g^−1^, while those of Co–ZIF-350 and Co–ZIF-550 were 30.6 m^2^ g^−1^ and 22 m^2^ g^−1^, respectively. The lowest surface area of Co–ZIF-550 marked the negative influence of nanocuboid aggregation.

**Figure 3:**
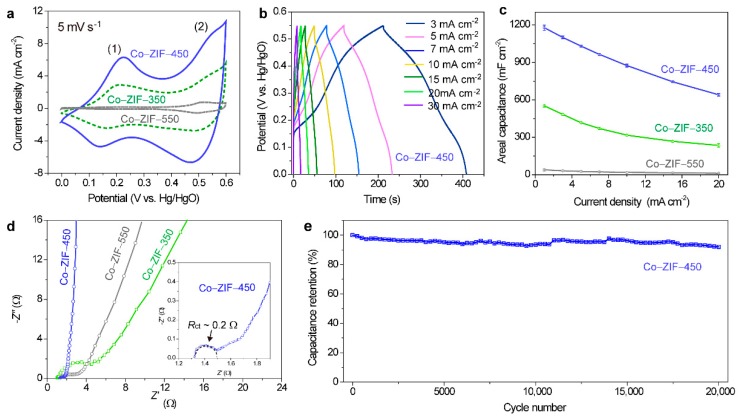
Electrochemical performances collected in 2 M aqueous KOH electrolytes via a three-electrode testing configuration. (**a**) Cyclic voltammetry (CV) curves of Co–ZIF-350, Co–ZIF-450, and Co–ZIF-550 at 5 mV s^−1^. (**b**) Galvanostatic charge and discharge curves of Co–ZIF-450 at different current densities. (**c**) Comparison of areal capacitances of Co–ZIF-350, Co–ZIF-450, and Co–ZIF-550 at 1–20 mA cm^−2^. The error bars are standard deviations determined at least in triplicate. (**d**) Nyquist plots of Co–ZIF-350, Co–ZIF-450, and Co–ZIF-550. Inset: Enlarged view of the high- to middle-frequency domains of the Nyquist plot of Co–ZIF-450. (**e**) Cycling stability of Co–ZIF-450 after 20,000 consecutive charge–discharge cycles.

Co–ZIF-450 exhibited the best electrochemical performance among all the tested samples. The enclosed area by the cyclic voltammetry (CV) curve of Co–ZIF-450 was appreciably larger than those of Co–ZIF-350 and Co–ZIF-550 (Figure 3a), indicating that Co–ZIF-450 had the largest capacitance. The two pairs in the CV curves, one within 0.1–0.2 V (vs. Hg/HgO) and another one at ~0.5 V (vs. Hg/HgO), resulted from the following redox reactions [15]:

3CoO + 2OH^−^ ↔ Co_3_O_4_ + H_2_O + 2*e*^−^ (2)

Co_3_O_4_ + OH^−^ + H_2_O ↔ 3CoOOH + *e*^−^ (3)

The broad redox peaks demonstrated pseudocapacitive behavior. Additionally, the pseudocapacitive characteristic of Co–ZIF-450 was reflected from the symmetric and plateau-free galvanostatic charge–discharge curves (Figure 3b). Among all the tested electrodes, Co–ZIF-450 achieved the highest areal capacitance of 1177 mF cm^−2^ at 1 mA cm^−2^ (Figure 3c), corresponding to a gravimetric capacitance of 370 F g^−1^ (based on the active mass of ~3.2 mg cm^−2^, Figure S4, Supplementary Materials). Remarkably, these values surpassed those of the previously reported cobalt oxide-based pseudocapacitive electrodes, such as MOF-derived dendrite-like Co_3_O_4_ nanomaterial (207.8 F g^−1^ @ 0.5 A g^−1^) [35], Co-based MOF (179 F g^−1^ @ 10 mV s^−1^) [36] and MOF-derived porous Co_3_O_4_ (150 F g^−1^ @ 1 A g^−1^) [37]. When the current density increased 20 times to 20 mA cm^−2^, Co–ZIF-450 still acquired an areal capacitance of 640 mF cm^−2^ (equivalent to 54.4% capacitance retention).

Co–ZIF-350 and Co–ZIF-550 were unable to compete with Co–ZIF-450. When the current density changed from 1 to 20 mA cm^−2^, the areal capacitance of Co–ZIF-350 decreased from 553 mF cm^−2^ to 235 mF cm^−2^, and that of Co–ZIF-550 dropped from 37 mF cm^−2^ to 11 mF cm^−2^. We speculated that Co–ZIF-350 suffered from the low electrical conductivity due to the incomplete decomposition of Co–ZIF. The electrochemical performance of Co–ZIF-550 was restricted by the structural collapse as discussed earlier.

The Nyquist plot of Co–ZIF-450 disclosed more insights into its electrochemical performance. The plot displayed a semi-circle in the high-frequency domain and a vertical spike in the low-frequency region (Figure 3d), resembling the plots of electrodes having typical capacitive behaviors [38]. Both the equivalent series resistance (*R*_s_, the intercept of the plot with *Z*’-axis) and the charge-transfer resistance (*R*_ct_, the diameter of the semi-circle) of Co–ZIF-450 are small (~1.3 Ω and ~0.2 Ω, respectively). The blending of electrically conductive carbon matrix and Co metal eased the electron transport in the electrode and accounted for the small *R*_s_. Additionally, the nanoporous nanocuboid arrays facilitated ion diffusion. This factor, together with the rapid electron transport, decreased *R*_ct_. The *R*_ct_ of Co–ZIF-350, Co–ZIF-450, and Co–ZIF-550 were about 4.0 Ω, 0.2 Ω, and 2.0 Ω, respectively. The relatively large *R*_ct_ of Co–ZIF-350 came from the incomplete decomposition that yielded little Co metal particles and carbon for fast electron conduction near the electrode|electrolyte interfaces. The *R*_ct_ of Co–ZIF-550 raised slightly, as a result of structural collapse that retarded the redox reactions involving electron–ion interactions (evidenced from the CV result).

The capacitance of Co–ZIF-450 was maintained at 93.5% after 10,000 repetitive charge–discharge cycles, which is comparably good than those of Co–ZIF-350 (95.6%) and Co–ZIF-550 (93.6%) after identical cycle numbers (Figure S5, Supplementary Materials). Significantly, the capacitance of Co–ZIF-450 was kept stable with no capacitance loss after 20,000 continuous charge–discharge cycles (Figure 3e).

Taking together, we ascribed the outstanding electrochemical performance of Co–ZIF-450 to four reasons: First, the metal Co and N-doped carbon constructed a highly conductive network which facilitated electron transport in the electrode. Second, the uniformly distributed pores in each nanocuboid acted as electrolyte ion reservoirs that shortened the diffusion distances to electrode surfaces and thus, facilitated ion diffusion. Third, the in-situ growth of nanocuboids eliminated the need for electrically insulating binders and reduced the contact resistance between the current collector (carbon cloth) and the active materials. Fourth, the soft carbon matrix effectively buffered the mechanical stress imposed by cobalt oxide during charge and discharge processes, leading to the durable charge-storage performance.

**Figure 4:**
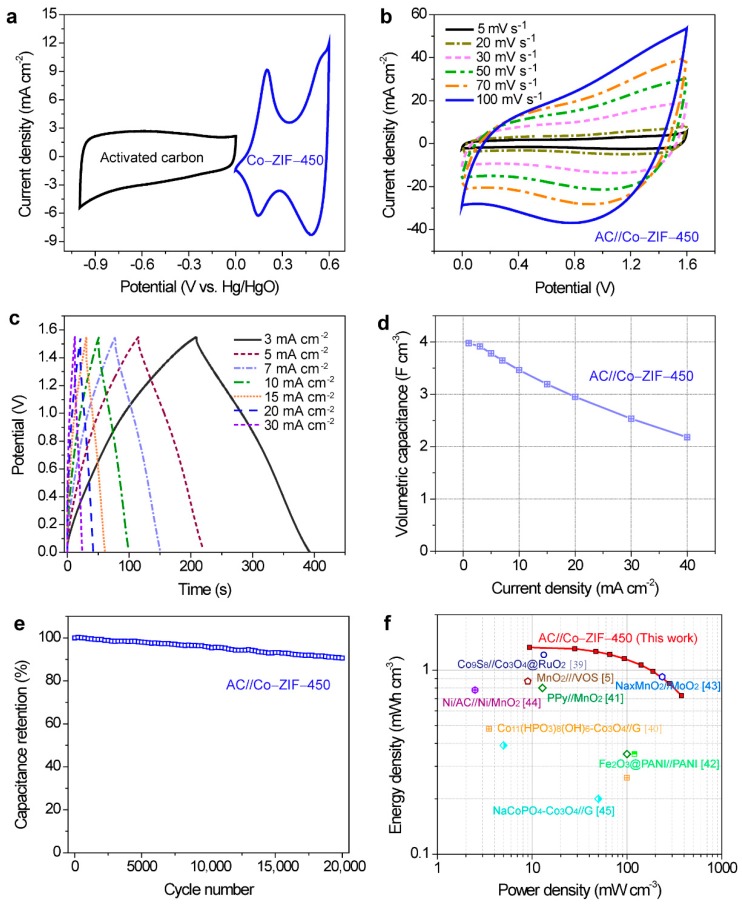
Electrochemical performances of the asymmetric supercapacitor prototype (AC//Co–ZIF-450) using 2 M aqueous KOH electrolytes. (**a**) CV curves of Co–ZIF-450 (positive) and activated carbon (negative) at different potential windows. (**b**) CV curves at scan rates from 5 to 100 mV s^−1^. (**c**) Galvanostatic charge and discharge curves at current densities from 3 to 30 mA cm^−2^. (**d**) Volumetric capacitances as a function of current density. (**e**) Cycling stability during 20,000 charge and discharge cycles. (**f**) The Ragone plots comparing the energy and power densities of our device to selected previously demonstrated asymmetric supercapacitors. The red line is a guide.

To evaluate the potential of Co–ZIF-450 under realistic working conditions, we assembled an asymmetric supercapacitor prototype using Co–ZIF-450 as the positive electrode and activated carbon powders (AC) coated on carbon cloth as the negative electrode (the electrochemical activities of the AC electrode is summarized in Figure S6, Supplementary Materials). The asymmetric device is denoted as AC//Co–ZIF-450. The stable potential windows of Co–ZIF-450 and activated carbon were 0 to 0.55 V (vs. Hg/HgO) and −1.0 to 0 V (vs. Hg/HgO), respectively (Figure 4a), adding up to a theoretical overall potential window of 1.55 V for AC//Co–ZIF-450. Indeed, AC//Co–ZIF-450 displayed CV curves devoid of a sharp increase in current at potentials up to 1.55 V (Figure 4b). Its cyclic voltammogram also enclosed larger area than those of AC//Co–ZIF-350 and AC//Co–ZIF-550 (Figure S7, Supplementary Materials), showing the highest capacitance (102, 288, 28.6 mF cm^−2^ for AC//Co–ZIF-350, AC//Co–ZIF-450, and AC//Co–ZIF-550, respectively). The symmetric galvanostatic charge and discharge curves suggested good Coulombic efficiency, and the sloping feature marked the capacitive nature of the prototype (Figure 4c). Outstandingly, our asymmetric device achieved a high volumetric capacitance of 4 F cm^−3^ at 1 mA cm^−2^ (Figure 4d), surpassing those of the state-of-the-art supercapacitors with similar thicknesses, including Co_9_S_8_ nanorod//Co_3_O_4_@RuO_2_ (3.42 F cm^−3^ @ 2.5 mA cm^−2^) [39], Co_11_(HPO_3_)_8_(OH)_6_-Co_3_O_4_//graphene (1.84 F cm^−3^ @ 0.5 mA cm^−2^) [40], and VO_x_//VN (1.35 F cm^−3^ @ 0.5 mA cm^−2^) [22]. The volumetric capacitance remained 2.15 F cm^−3^ at 40 mA cm^−2^. Besides, the prototype maintained ~91% of its initial capacitance after being subjected to 20,000 continuous charge–discharge cycles. In terms of energy-storage capacity, our prototype presented a volumetric energy density of 1.32 mWh cm^−3^ at a power density of 9.4 mW cm^−3^, and 0.73 mWh cm^−3^ at 376 mW cm^−3^, higher than supercapacitors with comparable volumes [5,39,40,41,42,43,44,45], e.g., Co_9_S_8_ nanorod//Co_3_O_4_@RuO_2_ (1.21 mWh cm^−3^ @ 13.29 mW cm^−3^) [39], NaCoPO_4_-Co_3_O_4_//graphene (0.39 mWh cm^−3^ @ 5 mW cm^−3^) [45], Co_11_(HPO_3_)_8_(OH)_6_-Co_3_O_4_//graphene (0.48 mWh cm^−3^ @ 3.5 mW cm^−3^) [40], and PPy//MnO_2_ (0.8 mWh cm^−3^ @ 12.85 mW cm^−3^) [41] (Figure 4f).

## 4. Conclusions

In summary, this work demonstrates the synthesis of cobalt-incorporated, N-doped nanoporous carbon nanocuboids via chemical bath deposition of Co–ZIF and thermal annealing at 450 °C. The highly conductive Co metal and N-doped carbon network facilitated electron transport within the electrode, and the nanopores of each carbon nanocuboid functioned as electrolyte reservoirs that reduced ion diffusion distance from the electrolyte to electrode surfaces. Both factors contributed to the promising charge–storage performance of Co–ZIF-450. This electrode exhibited an areal capacitance of 1177 mF cm^−2^ at the current density of 1 mA cm^−2^, which was 2 and 30 times higher than those of Co–ZIF-350 and Co–ZIF-550, respectively. Additionally, Co–ZIF-450 also displayed cycling stability with 94% capacitance retention after 20,000 consecutive charge–discharge cycles. By coupling with an activated carbon negative electrode, Co–ZIF-450 is a promising positive electrode in an asymmetric device prototype. The prototype delivered a volumetric energy density of 1.32 mWh cm^−3^ at the volumetric power density of 9.4 mW cm^−3^, outperforming other state-of-the-art supercapacitors with comparable volumes. This work highlights the functionality of metal–organic frameworks as precursors to highly porous metal oxide–carbon composite supercapacitor electrodes. These electrodes seamlessly integrate the merits of both pseudocapacitive and electrical double layer capacitive materials. Besides electrochemical energy storage demonstrated herein, we believe that the nanoporous Co–ZIF-based composites could be useful in a broad range of energy storage and conversion techniques, such as rechargeable batteries.

## Supplementary Materials

The following are available online at https://www.mdpi.com/2079-4991/9/8/1110/s1, Figure S1: A side-view SEM image of the Co–ZIF; Figure S2: (a) SEM image and (b) carbon, (c) nitrogen, (d) oxygen, and (e) cobalt elemental mappings of Co–ZIF-450. Scale bar in (a): 5 µm. Figure S3: (a) Calibration curve of solution absorbance with various MB concentrations. (b) UV-vis spectra collected for Co-ZIF-350, Co-ZIF-450, and Co-ZIF-550. Figure S4: Gravimetric capacitances of Co–ZIF-X as a function of current densities; Figure S5: Cycling stability performances of Co–ZIF-350 and Co–ZIF-550. Figure S6: (a) CV curve and (b) constant-current charge and discharge profiles of the AC negative electrode. Figure S7: Cyclic voltammograms of AC//Co–ZIF-350, AC//Co–ZIF-450 and AC//Co–ZIF-550 at 50 mV s^−1^.
