# Supplementary material for: Cobalt-Containing Nanoporous Nitrogen-Doped Carbon Nanocuboids from Zeolite Imidazole Frameworks for Supercapacitors
Source: Nanomaterials (Basel). 2019 Aug 2;9(8):1110. doi: 10.3390/nano9081110 (PMC6723694; doi:10.3390/nano9081110)
Supplement: Supplementary file 1 [file nanomaterials-09-01110-s001.pdf]

Communication

Cobalt-Containing Nanoporous Nitrogen-Doped Carbon Nanocuboids from Zeolite Imidazole Frameworks for Supercapacitors

Yu Song [1], Mingyue Zhang [1], Tianyu Liu [2], Tianjiao Li [1], Di Guo [1,*] and Xiao-Xia Liu [1,*]

[1] Department of Chemistry, Northeastern University, Shenyang 110819, China
[2] Department of Chemistry, Virginia Tech, Blacksburg, VA 24060, USA
* Correspondence: guodi@mail.neu.edu.cn (D.G.); xxliu@mail.neu.edu.cn (X.-X.L.)

Received: 8 July 2019; Accepted: 30 July 2019; Published: date

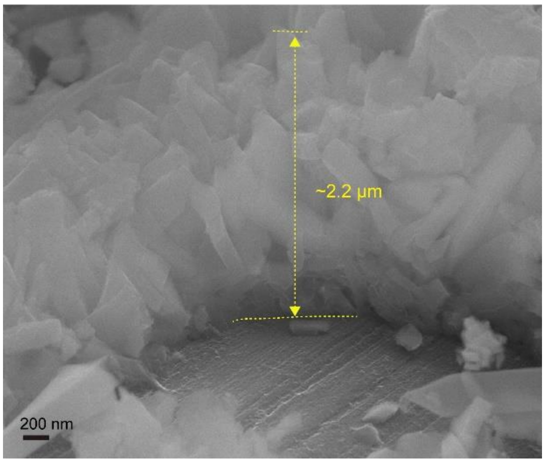

Figure S1. A side-view SEM image of Co–ZIF showing its thickness.

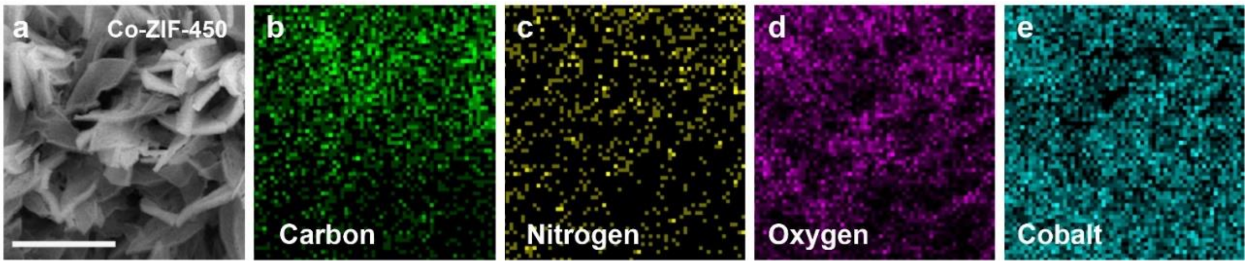

Figure S2. (**a**) SEM image and (**b**) carbon, (**c**) nitrogen, (**d**) oxygen, and (**e**) cobalt elemental mappings of Co–ZIF-450. Scale bar in (**a**): 5 μm.

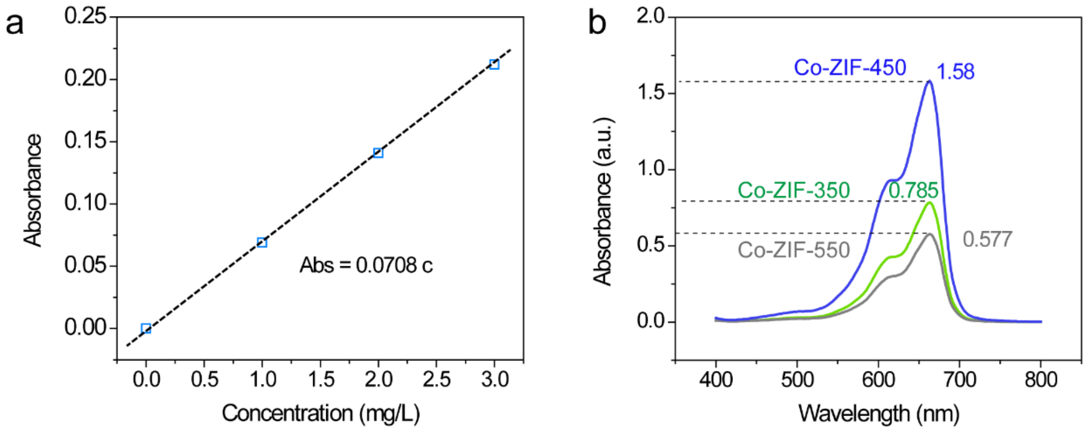

Figure S3. (**a**) Calibration curve of solution absorbance with various MB concentrations. (**b**) UV-vis spectra collected for Co–ZIF-350, Co–ZIF-450, and Co–ZIF-550.

The calibration curve was set up *via* measuring the absorbance of methylene blue (MB) aqueous solutions at the absorption wavelength of ~650 nm with concentrations spanning from 0.0 to 3.0 mg L^{-1} (Figure S3a). Equation S1 is the linear fitting line of the calibration curve:

$$A = 0.0708\ c \qquad (S1)$$

where A is absorbance, and c is the concentration of MB (mg L^{-1}). Afterward, a piece of sample (geometric area: 1 cm × 1 cm) was immersed in a 40 mg L^{-1} MB solution under constant shaking for 24 h. Then the sample was gently rinsed with de-ionized water to remove any residues that were loosely attached on the sample surface and dried in air for 3 h. The dried sample was immersed in 20 mL acetonitrile for 30 min to allow desorption of MB. At last, the absorbance of the resultant solution was measured by a UV-vis spectrometer (Agilent Cary 60). The highest absorbance at the maximal absorption wavelength (~650 nm) of Co–ZIF-450 reflected that it desorbed the largest amount of MB and therefore, it had the highest dye-accessible surface area at room temperature.

Moreover, we quantified the surface areas of the three materials using the MB absorption data. The concentrations of MB desorbed from electrode surfaces (denoted as c_{de}, in mg/L) were determined by substituting the absorbance at 650 nm into Equation (S1). Surface areas (S) was then estimated from Equation S2, [1,2]

$$S = \frac{c_{de} \times V \times 10^{-6} \times N_A \times S_{MB}}{M_{MB} \times m_{elec}} \qquad (S2)$$

where V is the volume of acetonitrile (20 mL); N_A is the Avogadro number (6.023×10^{23} mol^{-1}); S_{MB} is the molecular cross-sectional area of MB (120 Å or 120×10^{-20} m^2); M_{MB} is the molecular weight of MB (373.9 g mol^{-1}); m_{elec} is the mass of material used for the UV-vis measurement (14 mg or 0.014 g). The dye-adsorption surface area of Co–ZIF-450 reached 61.6 m^2 g^{-1}, while those of Co–ZIF-350 and Co–ZIF-550 were 30.6 m^2 g^{-1} and 22 m^2 g^{-1}, respectively. The lowest surface area of Co–ZIF-550 marked the negative influence of nanocuboid aggregation.

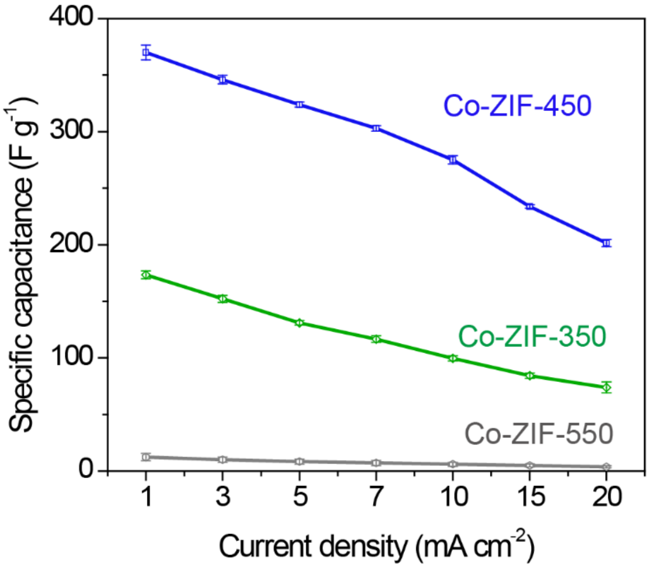

Figure S4. Gravimetric capacitances of Co–ZIF-X as a function of current densities. The error bars are standard deviations determined at least in triplicate.

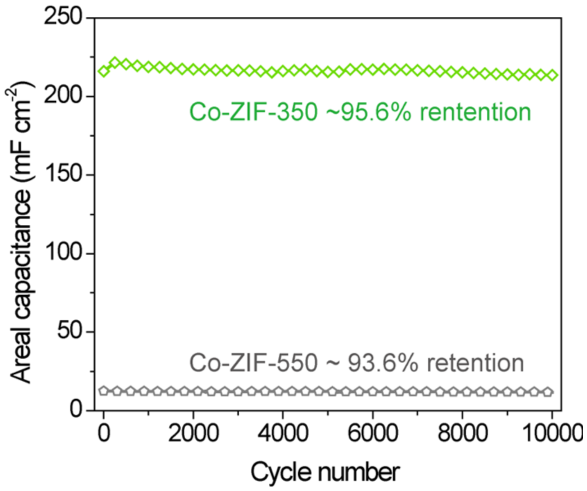

Figure S5. Cycling stability performances of Co–ZIF-350 and Co–ZIF-550.

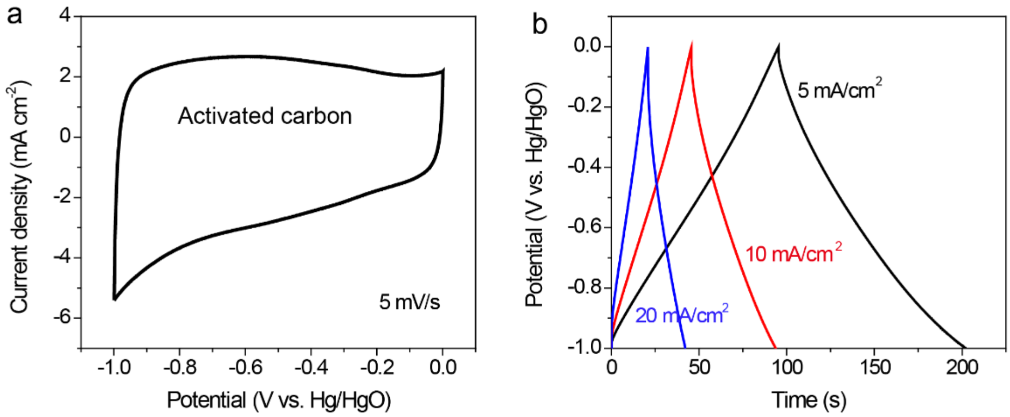

Figure S6. (**a**) CV curve and (**b**) constant-current charge and discharge profiles of the AC negative electrode.

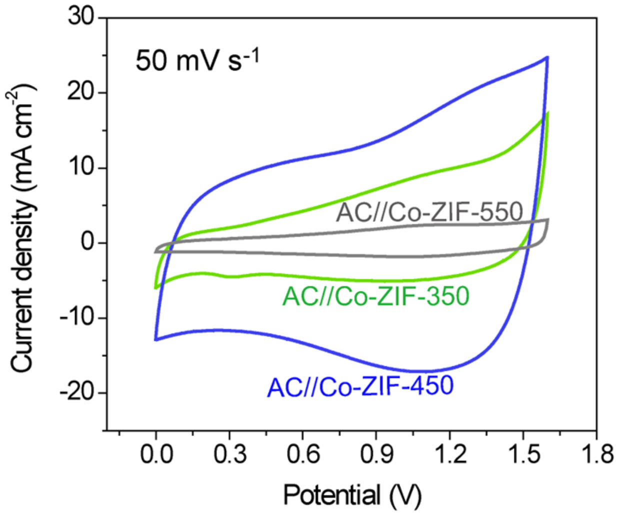

Figure S7. Cyclic voltammograms of AC//Co–ZIF-350, AC//Co–ZIF-450 and AC//Co–ZIF-550 at 50 mV s^{-1}.

References

1. Liu, T., Zhu, C., Kou, T., Worsley, M. A., Qian, F., Condes, C., Duoss, E., Spadaccini, C., Li, Y. Ion Intercalation Induced Capacitance Improvement for Graphene-Based Supercapacitor Electrodes. *ChemNanoMat*, 2016, 2(7), 635–641.
2. Wang, G., Wang, H., Lu, X., Ling, Y., Yu, M., Zhai, T., Tong, Y., Li, Y. Solid-state supercapacitor based on activated carbon cloths exhibits excellent rate capability. *Adv. Mater.*, 2014, 26(17), 2676–2682.
